# Adaptive functioning in school-aged children with spinal muscular atrophy in the treatment era: a non-randomised cohort study

**DOI:** 10.1016/j.lanwpc.2026.101866

**Published:** 2026-04-30

**Authors:** Emily E. Farah, Sarah-Grace Paguinto, Melissa R. Mandarakas, Karen Herbert, Smriti Krishan, Hugo Sampaio, Didu S. Kariyawasam, Michelle A. Farrar

**Affiliations:** a Discipline of Paediatrics and Child Health, School of Clinical Medicine, UNSW Medicine and Health, University of New South Wales, Sydney, Australia; b Department of Neurology, Sydney Children's Hospital Randwick, Sydney, Australia; c Occupational Therapy Department, Sydney Children's Hospital Randwick, Sydney, Australia; d Department of Psychology, Sydney Children's Hospital Randwick, Sydney, Australia; e Neuroscience Research Australia, UNSW Sydney, 2031, Australia

**Keywords:** Adaptive functioning, International classification of functioning, Patient-reported outcomes, Spinal muscular atrophy

## Abstract

**Background:**

Despite diagnostic and therapeutic advances for spinal muscular atrophy (SMA), long-term outcomes focused on functional independence for children have not been explored. This study aimed to characterise adaptive functioning for affected children within the contemporary SMA treatment paradigm.

**Methods:**

This prospective, non-randomised Australian cohort study was conducted from January 1 to November 14 2025 and included children with SMA aged 4–12 years, diagnosed and treated through newborn screening (NBS) or clinical referral (CR). Adaptive functioning was primarily assessed using the Pediatric Evaluation of Disability Inventory Computer Adaptive Test.

**Findings:**

Thirty-nine children participated (NBS n = 18, CR n = 21) with median treatment duration of 67.3 months (IQR 54.0–85.0). Children had high levels of participation with scores within expected range for age-matched peers for social/cognitive (38/39, 97%), responsibility (35/39, 90%), daily activities (29/39, 74%) and mobility (16/39, 41%) domains. A greater proportion of children identified through NBS achieved expected scores for all domains compared to children diagnosed through CR (NBS 14/18 (78%), CR 2/21 (10%), p < 0.001). All children diagnosed through NBS with 3 survival motor neuron 2 gene (*SMN2*) copies achieved expected scores for all domains (3 *SMN2* 7/7 (100%), 2 *SMN2* 7/11 (64%)). Children diagnosed through CR with higher functional status at diagnosis reported greater frequency of daily activities scores within expected range (walkers 7/7 (100%), sitters 2/5 (40%), non-sitters 3/9 (33%), p = 0.02).

**Interpretation:**

Whilst heterogenous in terms of performance-based measures, children with SMA have collective strengths in social-cognitive and responsibility aspects of adaptive functioning. Early diagnosis and treatment through NBS and *SMN2* copy number are important modifiers of long-term adaptive functioning, providing evidence for individualized goal-setting, monitoring and multidisciplinary care.

**Funding:**

National Health and Medical Research Council Investigator Grant (1194940, 2026317), University of New South Wales (UGCA1064).

**Research in context**

**Evidence before this study**

While the effectiveness of disease modifying therapies to improve survival, and motor and respiratory function has transformed the clinical course of spinal muscular atrophy (SMA), most clinical trials and real-world studies describe short term follow up. We conducted a PubMed search to evaluate the long-term treatment implications from January 1, 2015 to January 10, 2026 using search terms “spinal muscular atrophy”, AND “motor function”, AND “nusinersen”, “risdiplam”, or “onasemnogene abeparvovec”, AND/OR “adaptive function” AND/OR “functioning”. Results were filtered to only include articles that reported long-term data, with follow up >four years, in those who initiated treatment soon after birth or during childhood. Of the six articles, including two clinical trials and four real world studies, the outcomes measured were survival, the need for ventilatory support, improvements in motor function, and the occurrence of adverse drug reactions. For those who initiated treatment in the presymptomatic stage soon after birth, continued improvements in motor function and durability were reported. In treated symptomatic patients, improvements in motor function were maintained, and better in those with a shorter disease duration and higher motor function at baseline. While motor function differences persisted in treated children, their impact on adaptive functioning (daily activities and independence) and participation have not yet been described.

**Added value of this study**

This prospective study reports adaptive functioning (daily activities and independence) and participation in a heterogenous, real-world cohort of 39 children with SMA who have received disease modifying therapies. By combining patient-reported outcomes and performance-based measures, we provide an average of over five years (and up to ten years total) follow-up, with this extended interval capturing some of the first real-world longer-term outcomes of a newborn screening cohort who are school-aged. The findings show that adaptive functioning is diverse among children with SMA, and high levels of adaptive functioning are possible, particularly for children identified through newborn screening who receive prompt treatment. For children who can walk, longer distances, inclines and fatigue are important factors for daily independence. High levels of adaptive function were also possible for children treated symptomatically, facilitated by the presence of supportive environmental factors including assistive technologies and school supports to overcome functional challenges.

**Implications of all the available evidence**

Our results confirm that newborn screening for SMA coupled with disease modifying therapy yields significantly better longer-term outcomes. *SMN2* copy number and motor function at diagnosis are also important modifiers of long-term functional outcomes, informing individualised goal setting. Multidisciplinary care is important to meet the changing and individualised needs of children with SMA, especially as they transition to complete higher motor tasks and complex everyday activities. Health professionals and families must focus on skills and targeted interventions for promoting independence and participation at home, school and within the community.

## Introduction

Spinal muscular atrophy (SMA) is a genetic neuromuscular disorder characterised by progressive muscle weakness and atrophy, leading to accrual of disability. The condition has a wide spectrum of severity, with the most common and severe form previously known as the leading genetic cause of infant death.^1^ SMA is caused by biallelic pathogenic variants of exon 7 of the survival motor neuron 1 (*SMN1*) gene.^1^ The paralogous survival motor neuron 2 (*SMN2*) gene is a phenotypic modifier, with copy number inversely correlated with disease severity.^1^ The development of three disease modifying treatments (DMTs), advancements in standards of medical care and newborn screening (NBS) have not only improved survival but transformed the clinical trajectory for people with SMA. However, phenotypic heterogeneity is evident amongst individuals with SMA, with novel phenotypes emerging among children treated in early life.^2^

Clinical trials and real-world studies have utilised performance-based assessments focused on motor, respiratory and bulbar function outcomes, facilitating the translation of therapies into clinical practice. Findings indicate that DMTs are most effective when administered early, ideally before clinical signs or symptoms are evident.^1^ While the acquisition of motor milestones has been observed in infants treated over several years, knowledge of the therapeutic effects beyond these is limited, especially for those now surviving to early childhood with the severe, infantile onset form of SMA.

Furthermore, current evidence is limited in capturing the multidimensional effects of SMA upon the lives of affected people, their caregivers and families, with a paucity of data available for children emerging through NBS programs. For children with SMA, families and clinicians, the ability to thrive extends to everyday functioning, activities and participation at home, school and the community.^3^, ^4^, ^5^ Here the emphasis is on adaptive functioning, denoting an individual's capability to navigate their personal everyday needs and actively participate in their environment, aligning with standards of independence expected for the individual's age and environmental context.^6^

Few studies have explored adaptive functioning in small cohorts of children with SMA, predominantly as a secondary outcome amongst older symptomatic children initiating DMTs.^7^^,^^8^ Ongoing challenges in adaptive functioning were described in these populations, with improvements centred on upper limb function. Importantly, these studies have not yet captured adaptive functioning in children diagnosed and treated through new diagnostic pathways, namely NBS. Furthermore, a knowledge gap exists in understanding adaptive functioning using person-reported outcome measures to holistically capture meaningful changes.

To bridge these gaps, the aim of this study was to characterise adaptive functioning and associated factors of school-aged children with SMA who received DMTs through NBS and clinical referral pathways, using person/caregiver-reported outcome measures. The secondary aims were to evaluate motor function trajectories of children with SMA who received DMTs and explore necessary functional supports and environmental influences affecting adaptive functioning.

## Methods

### Study design

This was a single-centre non-randomised cohort study. The study design was informed by results from a previous study, where significant differences in motor function, non-invasive ventilation and feeding support in children according to modality of diagnosis were established at two years post-diagnosis.^2^

### Study setting, population and recruitment

This study was conducted from January 1 to November 14 2025 within the Sydney Children's Hospital Network (SCHN), a quaternary referral centre for children with SMA. Eligibility criteria included children with genetically confirmed 5q-SMA who had received DMTs, and were transitioning to, or attending, primary school (age ≥4 years). Children were consecutively and non-selectively assigned to two subgroups: diagnosis through NBS or clinical referral (CR). Children with a separate diagnosis that could independently affect adaptive functioning (e.g., cerebral palsy, concurrent genetic disorder), or who were receiving palliative care were excluded. Children were identified through the SCHN neuromuscular database and study information was provided by email prior to appointments. Participants were recruited during hospital visits and all caregivers provided written informed consent. A certified interpreter assisted non-English speaking participants.

### Ethics approval

Ethical approval was granted by the Sydney Children's Hospital Network Human Research Ethics Committee [2022/ETH01937]. All caregivers provided written informed consent to participate.

### Measures

Study measures were selected based on the World Health Organization's International Classification of Functioning, Disability and Health (ICF). This considers how health conditions, body structures and contextual factors influence an individual's ability to complete everyday activities and participate (Supplementary Figure S1).^9^ Sociodemographic and clinical characteristics were collected from medical records, including age, sex, *SMN2* copy number, age at diagnosis and treatment, and functional status at treatment initiation and study enrolment.^10^

The primary outcome measure was parent and caregiver outcomes as evaluated by the English version of the Pediatric Evaluation of Disability Inventory Computer Adaptive Test (PEDI-CAT), validated for children with SMA, measuring activities and participation.^11^, ^12^, ^13^ This was piloted with caregivers of children with a range of modalities of diagnosis and *SMN2* copy numbers to evaluate format, content suitability and score fit. Designed according to item response theory, the PEDI-CAT uses artificial intelligence to select relevant items assessing everyday activities and participation from a validated bank across four domains: daily activities (approximately 30 items), mobility (approximately 30 items), social/cognitive (approximately 15 items), and responsibility (approximately 15 items), each item representing different levels of difficulty to perform.^13^ The primary caregiver was asked to rate their child's typical performance (“unable”, “hard”, “a little hard”, “easy”, “I don't know”), generating a scaled and normative score via the QGlobal platform (Pearson Clinical, Bloomington, USA). Scaled scores (range 20–80 points, indicative of a continuum of performance) provide an objective measure of typical daily functioning, and normative scores (range <10–90) enable a comparison of functioning to age-matched peers, where higher scaled and normative scores are indicative of higher function. For children with a diagnosis of autism spectrum disorder (ASD), an ASD PEDI-CAT module was administered, including revised items and adjusted scaling of the social/cognitive domain. For children who used a manual wheelchair, a wheelchair sub-domain option within the mobility domain was administered.

Secondary outcome measures included motor function assessments conducted by a physiotherapist to measure body structures and functions and activities. These comprised the Hammersmith Functional Motor Scale—Expanded (HFMSE) (33 items, maximum 66 points) and Revised Upper Limb Module (RULM) (19 items, maximum 37 points), which are SMA-specific, criterion-referenced performance-based scales where higher scores are indicative of better function.^14^^,^^15^ Timed functional tests were undertaken in “walkers” to assess walking speed and fatigue, and included the Ten-Metre Walk/Run Test (10MWRT) and Six-Minute Walk Test (6MWT).^16^^,^^17^ These tests were conducted barefoot without assistive technology (e.g., ankle-foot orthoses). The fastest time of two 10MWRTs was recorded. The 6MWT distance walked was recorded, also expressed as the percent of the normative predicted 6MWT for age, height and weight.^18^ The Spinal Muscular Atrophy Functional Composite Score Revised (SMA-FCR) was used as a measure to broaden the spectrum of abilities captured in SMA, where higher scores are indicative of better function.^19^ This was calculated for each child using the weighted average of the HFMSE, RULM and percent of the normative predicted 6MWT performance as shown below.^19^ Here, non-ambulant children scored “0 m” for the 6MWT distance.

$$SMA-FCR=\left(\frac{HFMSE}{66}\;+\;\frac{RULM}{37}\;+\;\frac{6MWT\;Distance}{Predicted\;6MWT\;Distance}\right)\;\times \;\frac{100}{3}$$

Environmental factors measured included the Parental Stress Scale (18 items), administered by a psychologist in person or via telephone within three months of functional study assessments. This survey evaluated stress as pertaining to the care of their child with SMA, with caregivers self-reporting feelings about their parenting, including positive (e.g., emotional benefits) and negative aspects (e.g., demand on resources) on a Likert scale from 1 (strongly disagree) to 5 (strongly agree).^20^ Responses were summated ranging from 18 (low stress) to 90 (high stress). All caregivers were offered support information and resources by the psychologist. The use of assistive technologies and supports to complete daily tasks at home or school was also collated in a clinical survey (Appendix 1).

### Data analysis

Descriptive statistics were used to report clinical characteristics, caregiver-reported outcome measures, motor function outcomes and caregiver stress, with SPSS Version 27. For categorical variables, frequencies and percentages were reported, and for continuous variables medians and interquartile ranges (IQR). Normality was determined using the Shapiro–Wilk test. Differences in clinical characteristics and outcomes according to modality of diagnosis were tested for statistical significance using Chi-Square (χ^2^), Fisher's exact, Mann–Whitney U and Kruskal–Wallis tests as appropriate. The Bonferroni adjustment was applied for post hoc pairwise comparisons. Odds ratios (OR) with 95% confidence intervals (CI) were calculated for binary outcomes. Analyses were conducted using pairwise deletion, whereby all available data were used for each analysis. Statistical significance was defined as p < 0.05 (two-tailed). Normative scores (t-score and age percentile) were used to compare functioning to age-matched peers, with PEDI-CAT t-scores between 30 and 70 (i.e., mean ±2 standard deviations) considered within what is typically expected for the respective age range. Excel (Version 16.68, Microsoft Corporation) was used to collate PEDI-CAT item responses and functional supports. Graphical output was produced using Graphpad Prism by Dotmatics at graphpad.com, Version 10.2.0 (2024).

### Participatory engagement

Following data analysis, caregivers were invited via email to attend an online or in-person post-hoc consultation to share and discuss data, contextualising findings within a child and family-centred perspective.

### Role of the funding source

The funders did not have any role in study design, data collection, data analysis, interpretation, or writing of this report.

## Results

### Sociodemographic and clinical characteristics

Of 43 eligible children, 39 participated in the study (response rate 39/43, 91%) with a median DMT duration of 67.3 months (IRQ 54.0–85.0). Of this cohort 18/39 (46%) were diagnosed through NBS and 21/39 (54%) through clinical referral. The median age of children was 6.8 years (IQR 5.3–9.0), with those identified through NBS younger at the time of diagnosis, initiation of DMTs and study enrolment (Table 1). Two children had a diagnosis of ASD.

**Table 1:** Demographic, genetic and clinical characteristics of children with SMA.

	Newborn screening group (n = 18)	Clinically referred group (n = 21)	p value
Sex			
Male	7 (39%)	10 (48%)	0.748
Female	11 (59%)	11 (52%)	
Age			
Current age (years)	5.2 (4.5–5.9)	9.0 (8.3–10.4)	<0.001
Age at diagnosis (months)	0.5 (0.4–0.6)	12.0 (6.7–36.7)	<0.001
Age at DMT initiation (months)	0.8 (0.7–1.1)	27.0 (10.0–56.0)	<0.001
*SMN2* copy number			
2 copies	11 (59%)	6 (29%)	0.041^b^
3 copies	7 (39%)	13 (62%)	
4 copies	0	2 (10%)	
Functional status at diagnosis^a^			
Non-sitter	–	9 (43%)	–
Sitter	–	5 (24%)	–
Walker	–	7 (33%)	–
Clinical status at diagnosis			
Clinically manifest	4 (22%)	20 (95%)	<0.001
Clinically silent	14 (78%)	1 (5%)	
Current functional status^a^			
Non-sitter	0	1 (5%)	<0.001
Sitter	1 (6%)	13 (62%)	
Walker	17 (94%)	7 (33%)	
Assistive technology use			
Lower limb orthoses	9 (50%)	15 (71%)	0.203
Wheelchair (Manual/Power)	5 (28%)	16 (76%)	0.002
School supports			
No school supports	11 (59%)	5 (24%)	0.018^c^
Any school support	7 (39%)	16 (76%)	
Physical transfers, toileting	4 (22%)	13 (62%)	
Learning support	2 (11%)	2 (10%)	
Mobility supervision	1 (6%)	2 (10%)	

Data from included children (n = 39) are reported as median (interquartile range) or number (%).

Abbreviations: DMT, disease modifying therapies; SMA; spinal muscular atrophy; *SMN2*, survival motor neuron 2 gene.

a Evaluated by the highest motor skill achieved using the World Health Organization Multicentre Growth Reference Study.^10^

b χ^2^ calculated for 2 *SMN2* copies versus ≥3 *SMN2* copies.

c χ^2^ calculated for any support versus no support.

For children identified through NBS, 11/18 (61%) had 2 *SMN2* copies, of whom 4/11 (36%) were clinically manifest at treatment initiation. There was a greater proportion of children with ≥3 *SMN2* copies within the clinically referred group (NBS 7/18 (39%), CR 15/21 (71%), OR = 4.0 (95% CI 1.06–14.29), p = 0.041), in which all walkers (7/21, 33%) had ≥3 *SMN2* copies. Following initiation of DMTs there were more current walkers among children identified through NBS (NBS 17/18 (94%), CR 7/21 (33%), OR = 34.00 (95% CI 3.82–376.70), p < 0.001).

### Parent/caregiver reported outcomes as assessed by the PEDI-CAT

The PEDI-CAT was piloted with children and caregivers (n = 3), who supported its format and felt that it provided meaningful information regarding their child's adaptive function. The fit scores were within optimum ranges for daily activities (range −0.12 to 0.41), social/cognitive (range −0.13 to 1.21) and responsibility (range −1.52 to 1.3) domains, and less defined for mobility (range −2.31 to −1.71).

**Fig. 1:**
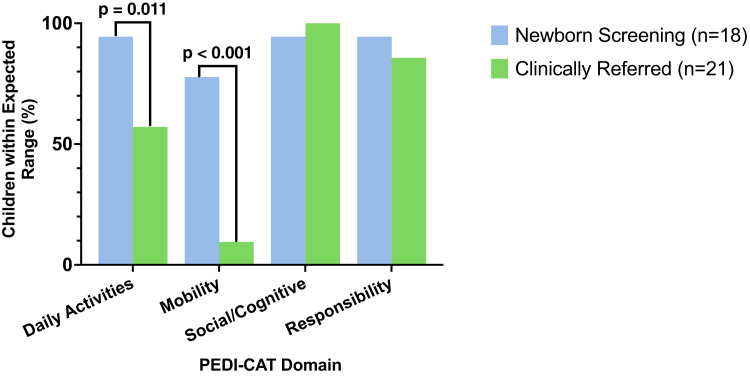
**Percentage of children with SMA reaching expected PEDI-CAT scores for their age by modality of diagnosis.** Children (n = 39) were identified through newborn screening (blue) and clinical referral (green). Two children completed the ASD PEDI-CAT module for the social/cognitive domain; one child achieved a score within expected range. Abbreviations: PEDI-CAT, Pediatric Evaluation of Disability Inventory Computer Adaptive Test; SMA, spinal muscular atrophy.

Of the cohort, 16/39 (41%) of children had scores within the typically expected range for all PEDI-CAT domains. A greater proportion of children identified through NBS achieved scores within typically expected ranges for all four PEDI-CAT domains, in comparison to those diagnosed through clinical referral (NBS 14/18 (78%), CR 2/21 (10%), OR = 33.25 (95% CI 4.90–167.40), p < 0.001) (Fig. 1).

Adaptive strengths included social/cognitive (38/39, 97%) and responsibility (35/39, 90%) domains, with most children scoring within expected ranges and no differences between modality of diagnosis (social/cognitive NBS 17/18 (94%), CR 21/21 (100%), p = 0.462; responsibility NBS 17/18 (94%), CR 18/21 (86%), p = 0.609). All children diagnosed through clinical referral (21/21, 100%) achieved social/cognitive scores within expected range. Scores within the typically expected range were reported amongst 29/39 (74%) children in daily activities, and 16/39 (41%) children in the mobility domains. Here, a greater proportion of children diagnosed through NBS reported scores within the expected range for mobility (NBS 14/18 (78%), CR 2/21 (10%), OR = 33.25 (95% CI 4.90–167.40), p < 0.001) and daily activities (NBS 17/18 (94%), CR 12/21 (57%), OR = 12.75 (95% CI 1.84–146.70), p = 0.011) domains, compared to the clinically referred group. For children with daily activity and mobility scores below expectations, scaled scores were within the first centile of scores for 7/10 (70%) and 17/23 (74%) children respectively, and only one of these children was identified through NBS.

In domains demonstrating variations from expected norms (i.e., daily activities, mobility), a range of strengths and challenges were noted between the NBS and clinically referred groups (Tables 2 and 3). Functional strengths for the NBS group included independent ambulation and anti-gravity shoulder movements for self-care tasks, compared to standing for brief periods and whole hand grasping movements for the clinically referred group. Challenges to independent functioning included fine motor dexterity skills and using stairs without a handrail for the NBS subgroup with 2 *SMN2* copies, and anti-gravity upper limb movements, independent ambulation and toileting for the clinically referred group. For the 12 children who were manual wheelchair users, strengths included independent use of their wheelchair, while challenges included managing kerbs and self-propelling for extended periods.

**Table 2:** Strengths and challenges reported through the PEDI-CAT for children with SMA according to modality of diagnosis (N = 39).

PEDI-CAT domain	Response	Newborn screening group (n = 18)		Clinically referred group (n = 21)	
		Task	Number (%)	Task	Number (%)
Daily activities	“Easy/Little hard”	Fine motor tasks for feeding	17 (94%)	Grasping items with the whole hand for feeding	18 (86%)
		Tucking in a shirt	16 (89%)	Fine motor tasks for feeding (e.g., opening snack food)	14 (67%)
		Shoulder movements for self-care (e.g., bathing)	15 (83%)	Fine motor manipulation tasks for dressing (e.g., buttons)	13 (62%)
	“Hard/Unable”	Fine motor manipulation (e.g., tying shoelaces)	9 (50%)	Anti-gravity shoulder movements for self-care (e.g., drying hair with towel)	11 (52%)
		Cutting thick material with scissors (e.g., hard plastic on toys)	8 (44%)	Strength-based fine motor tasks (e.g., cutting food with fork and knife)	9 (43%)
		Putting on a front-buttoning shirt	7 (39%)	Independent toileting (e.g., wiping)	8 (38%)
Mobility	“Easy/Little hard”	Car transfers	16 (89%)	Sitting in an adult chair for a few minutes	14 (67%)
		Independent standing for a few minutes	15 (83%)	Manual wheelchair mobility/managing brakes	10 (48%)
		Standing from a chair unaided	13 (72%)	Walks and carries a full glass without spilling/manages blanket and pillows independently at bedtime	8 (38%)
	“Hard/Unable”	4-wheel drive/van transfers	8 (44%)	Independent standing for a few minutes without aids	13 (62%)
		Completing stairs without handrails	6 (33%)	Standing from the floor unaided	12 (57%)
		Walking extended distances/walking carrying a weight (e.g., backpack)	5 (28%)	Turn head to both sides lying on back	8 (38%)

The most commonly reported tasks identified by caregivers, rated as “Easy/Little Hard” or “Hard/Unable” for the daily activities and mobility PEDI-CAT domains.

Abbreviations: PEDI-CAT, Pediatric Evaluation of Disability Inventory Computer Adaptive Test; SMA, spinal muscular atrophy.

**Table 3:** Strengths and challenges reported through the PEDI-CAT for children with SMA who are manual wheelchair users (N = 12).

PEDI-CAT domain	Response	Task	Number (%)
Wheelchair use	“Easy/Little hard”	Independent use in home	12 (100%)
		Using wheelchair brakes	12 (100%)
		Moving quickly indoors	8 (66%)
	“Hard/Unable”	Managing kerbs	10 (83%)
		Self-propelling for extended periods	7 (58%)
		Self-propelling on ramps	6 (50%)
		Self-propelling outdoors	6 (50%)
Transfers	“Hard/Unable”	Transfers from wheelchair to other chairs	6 (50%)
		Transfers from floor to wheelchair	5 (42%)

The PEDI-CAT wheelchair module was administered to children with access to manual wheelchairs, including those with access to both manual and electric wheelchairs.The most commonly reported tasks identified by caregivers of children who use manual wheelchairs (n = 12), rated as “Easy/Little Hard” or “Hard/Unable”.

Abbreviations: PEDI-CAT, Pediatric Evaluation of Disability Inventory Computer Adaptive Test; SMA, spinal muscular atrophy.

#### PEDI-CAT outcomes in relation to SMN2 copy number and motor status at diagnosis

**Fig. 2:**
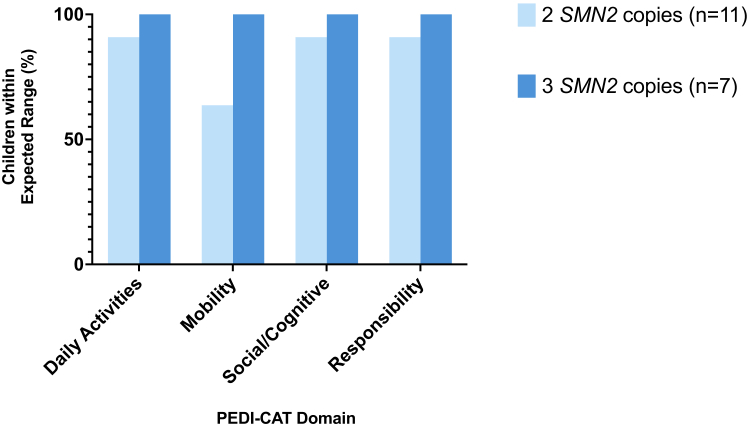
**Percentage of children with SMA identified through NBS reaching expected PEDI-CAT scores for their age as a determinant of *SMN2* copy number.** Children (n = 18) identified through NBS comprised 2 *SMN2* copies (light blue; n = 11) and 3 *SMN2* (dark blue; n = 7). Abbreviations: NBS, newborn screening; PEDI-CAT, Pediatric Evaluation of Disability Inventory Computer Adaptive Test; SMA; spinal muscular atrophy; *SMN2*, Survival Motor Neuron 2 gene. There were no children in the NBS group with 4 *SMN2* copies.

Within the NBS subgroup, all children with 3 *SMN2* copies (7/7, 100%) and 7/11 (64%) with 2 *SMN2* copies reported scores within expected range across all PEDI-CAT domains (Fig. 2). Of the four children who reported a mobility score outside the expected range, all had 2 *SMN2* copies (4/4, 100%) and 3/4 (75%) were clinically manifest at time of treatment.

Among children diagnosed through clinical referral, 7/7 (100%) walkers at diagnosis reported daily activities scores within the expected range, 2/5 (40%) sitters at diagnosis and 3/9 (33%) non-sitters at diagnosis (p = 0.020). Of children who were walkers at time of diagnosis, 2/7 (29%) reported mobility scores within the expected range, while this was achieved for 0/5 (0%) sitters and non-sitters (0/9, 0%) at diagnosis.

### Motor function outcomes

**Fig. 3:**
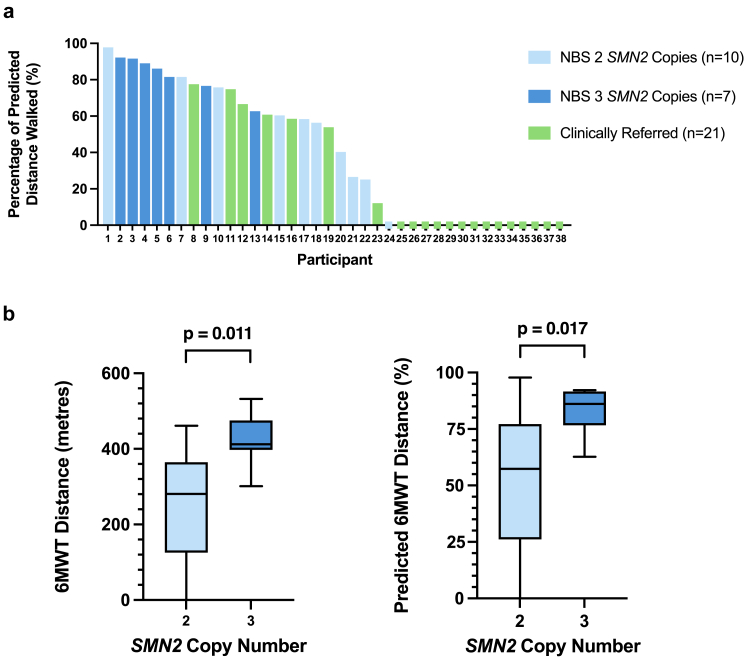
**6MWT outcomes achieved by children with SMA. a. Predicted distance walked by children with SMA in the 6MWT according to modality of diagnosis and *SMN2* copy number**. Waterfall plot, with each bar representative of one child (n = 38)∗ diagnosed through clinical referral (green), or newborn screening (blue). NBS children were further identified by 2 *SMN2* copies (light blue) or 3 *SMN2* copies (dark blue). The normative predicted distance was calculated for the child's age, height and weight using the STEP-IN SMA calculator, derived from the 1000 Norms Project.^18^ Children unable to stand or initiate the 6MWT scored “0”. **∗**One child from the NBS group did not comply with test protocols. **b. 6MWT distances achieved by children with SMA diagnosed through NBS, according to SMN2 copy number**. Box plots with bars representative of motor function scores of children (n = 17)∗∗ with 2 *SMN2* copies (light blue) or 3 *SMN2* copies (dark blue). Children unable to stand or initiate the 6MWT scored “0”. ∗∗One child with 2 *SMN2* copies did not comply with test protocols. Abbreviations: NBS, newborn screening; SMA; spinal muscular atrophy; *SMN2*, Survival Motor Neuron 2 gene; 6MWT, Six-Minute Walk Test. There were two children in the clinically referred group with 4 *SMN2* copies and no children in the NBS group had 4 *SMN2* copies.

Motor function measures and timed tests demonstrated variability according to modality of diagnosis (Supplementary Table S1). Higher HFMSE, 6MWT and SMA-FCR scores were reported in the NBS group compared to the clinically referred group (HFMSE, NBS median 63 (IQR 51–65.5), CR median 38 (IQR 14–57.5), p = 0.001; 6MWT, NBS median 350.0 m (IQR 236.0–417.0), CR median 0 m (IQR 0–323.0), p = 0.003; SMA-FCR, NBS median 79.75 (IQR 68.5–93.3), CR median 45.1 (IQR 29.5–85.4), p = 0.002). Despite the NBS group being younger, RULM scores showed no evidence of difference between groups (NBS median 30 (IQR 27–36), CR median 28 (IQR 21–37), p = 0.623). Nor was there evidence of a difference in time to complete the 10MWRT between walkers in the NBS and clinically referred groups (NBS median 5.6 s (IQR 4.3–7.8), CR median 3.6 s (IQR 3.3–8.2), p = 0.082), although a greater proportion of the NBS group completed this assessment (NBS 16/18 (89%), CR 7/21 (33%), OR = 16.0 (2.82–78.74), p < 0.001). The percentage of the predicted distance walked over 6 min demonstrated variation between modality of diagnosis (Fig. 3).

#### Motor function in relation to SMN2 copy number and motor status at diagnosis

**Fig. 4:**
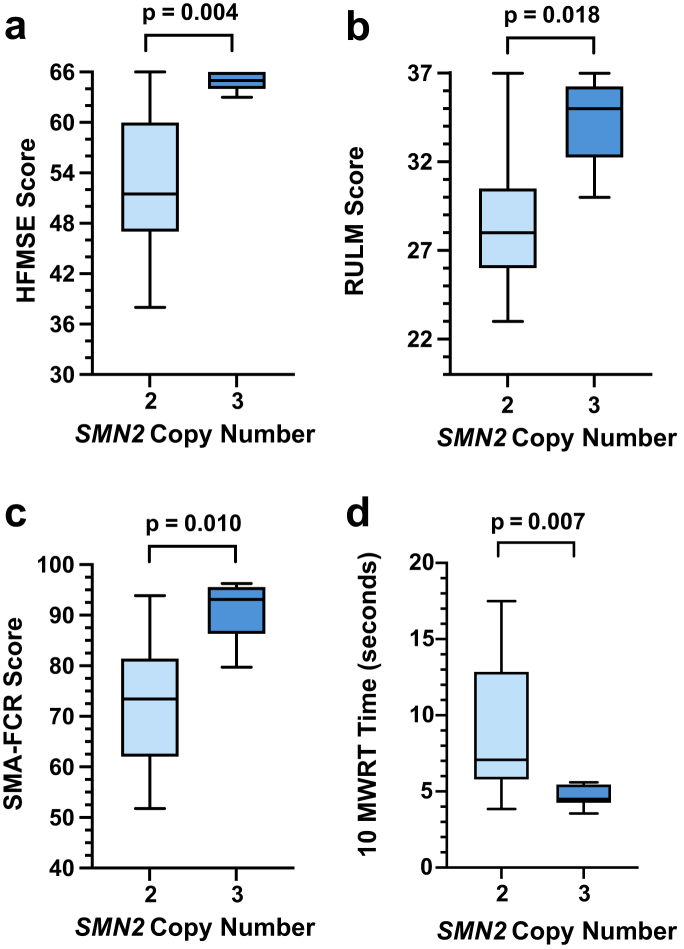
**Motor function scores achieved by children with SMA diagnosed through NBS, according to *SMN2* copy number**. **a.** Hammersmith Functional Motor Scale—Expanded scores **b.** Revised Upper Limb Module **c.** Spinal Muscular Atrophy Functional Composite Score Revised **d.** Ten-metre walk/run test. Box plots with bars representative of motor function scores of children (n = 18)∗ with 2 *SMN2* copies (light blue) or 3 *SMN2* copies (dark blue). ∗All motor function measures were collated for 15 children–2 children with 2 *SMN2* copies could not comply with the performance-based assessments, RULM missing for 2 children due to resource limitations. There were no children in the NBS group with 4 SMN2 copies.

A subgroup analysis of children diagnosed through NBS by *SMN2* copy number was completed (Fig. 4, Supplementary Table S2). Children with 3 *SMN2* copies reported a higher median score across all motor function tests compared to children with 2 *SMN2* copies. Time to complete the 10MWRT was faster for walkers with 3 *SMN2* copies compared to walkers with 2 *SMN2* copies (2 *SMN2* median 7.1 s (IQR 5.8–12.9), 3 *SMN2* median 4.5 s (IQR 4.3–5.5), p = 0.007).

Within the clinical referral group, children who were walkers at time of diagnosis reported higher scores for all motor function measures, compared to children who were sitters or non-sitters at the time of diagnosis (Table 4).

**Table 4:** Motor function scores achieved by children with SMA identified through clinical referral, as determined by functional status at diagnosis.

	Non-sitters (n = 9)	Sitters (n = 5)	Walkers (n = 7)	p value
HFMSE				
Median (IQR)	14 (8.5–35.5)	38 (13.5–44.5)	63 (53–65)	0.004^a^
Range	4–51	2–46	38–66	
RULM Dominant Hand				
Median (IQR)	23 (17.75–28.0)	27.5 (22.25–35)	37 (37–37)	0.007^b^
Range	11–30	21–37	27–37	
6MWT (metres)				
Median (IQR)	0 (0–0)	0 (0–0)	402 (78–452)	0.001^c^
Range	0–307	0–0	0–484	
Predicted 6MWT (metres)				
Median (IQR)	0 (0–0)	0 (0–0)	60.8 (12.1–74.8)	0.001^d^
Range	0–53.9	0–0	0–77.5	
SMA-FCR				
Median (IQR)	27.5 (19.3–43.9)	47.25 (34.9–51.7)	86.17 (64.1–91.0)	0.006^e^
Range	17.0–71.0	32.0–53.0	38.0–91.0	

Children (n = 21) were identified through clinical referral. RULM missing for two children due to resource limitations. Functional status determined by highest motor function at time of diagnosis. Scores represented in table reflect current motor function. Children unable to stand or initiate the 6MWT scored “0”. Between group comparisons conducted with post hoc pairwise comparisons with Bonferroni adjustment, significant at p < 0.05.

Abbreviations: HFMSE, Hammersmith Functional Motor Scale—Expanded; IQR, Interquartile Range; RULM, Revised Upper Limb Module; SMA, spinal muscular atrophy; SMA-FCR, Spinal Muscular Atrophy Functional Composite Score Revised; 6MWT, Six-Minute Walk Test.

a Non-sitters and walkers differed (adjusted p = 0.004) with a higher mean rank for walkers (walker mean rank = 17.14, non-sitter mean rank = 7.06).

b Non-sitters and walkers differed (adjusted p = 0.005), with a higher mean rank for walkers (walker mean rank = 14.86, non-sitter mean rank = 5.94).

c Non-sitters and walkers differed (adjusted p = 0.004), with a higher mean rank for walkers (walker mean rank = 16.79, non-sitter mean rank = 8.44), sitters and walkers differed (adjusted p = 0.007), with a higher mean rank for walkers (walker mean rank = 16.79, sitter mean rank = 7.50).

d Sitters and walkers differed (p = 0.007), with a higher mean rank for walkers (walker mean rank = 16.79, sitters mean rank = 7.50), non-sitters and walkers differed (adjusted p = 0.004), with a higher mean rank for walkers (walker mean rank = 16.79, sitter mean rank = 7.5).

e Non-sitters and walkers differed significantly (adjusted p = 0.004), with a higher mean rank for walkers (walkers mean rank = 15.0, non-sitter mean rank = 5.75).

### Environmental factors

Overall, more than one third of children (15/39, 38%) did not use assistive technologies or physical assistance to complete everyday tasks at home or school, of whom the majority (10/15, 67%) were diagnosed through NBS. All learning and mobility support (see Table 1) was offered in the context of mainstream educational settings, with government-funded personal support.

The parental stress survey was completed by 33 caregivers, and 15/33 (45%) had children diagnosed through NBS. The median total stress score was 40 (IQR 32–48), and no caregiver reported an elevated stress score above 55. Among respondents, there was no evidence of a difference in scores based on modality of diagnosis (NBS median 39 (IQR 32–48), CR median 41.5 (IQR 32–48), p = 0.799). Positive aspects of caregiving were endorsed, with all (28/28, 100%) agreeing or strongly agreeing that they enjoyed spending time with their children, their children were an important source of affection and enjoyment, and they were happy in their role as a parent (Supplementary Figure S2). Negative aspects of caregiving were reported with 15/33 (45%) caregivers agreeing or strongly agreeing that they were overwhelmed by the demands of being a parent, and 13/33 (39%) agreeing or strongly agreeing that the major source of stress in their life was their children.

### Participatory engagement

A summary of results was presented to five caregivers (four mothers, one father) after data analysis, of whom four had children diagnosed through NBS. All participants agreed the abilities and challenges elicited through the PEDI-CAT were reflective of their child's adaptive function and provided useful information for preparing relevant supports for their child, in particular transition to school. Caregivers endorsed the reporting of normative scores to provide a measure of typical daily functioning and comparison to age-matched peers. Furthermore, they were able to draw associations between environmental supports and level of adaptive functioning for their child. Parents valued how PEDI-CAT questions focused on real-world tasks and supported family-centred goals, which could be subsequently used as evidence for multidisciplinary interventions and funding applications.

## Discussion

The first cohort of children identified by NBS programs and accessing DMTs internationally are now reaching school age. Thus, the real-world evaluation of the commonalities and differences in their adaptive functioning and participation in daily life has become highly relevant. This is especially important as adaptive functioning has been under-evaluated thus far, with clinical trials and real-world studies instead concentrating upon performance-based motor function outcomes. These have demonstrated heterogeneity based upon modality of diagnosis, clinical status at treatment, and genotype, but have not informed a clinical model of care that can predict and ameliorate the individual challenges that affected children face as they navigate their home, social and educational environments. Our study provides extended follow up and addresses these gaps by identifying key modifiers of adaptive functioning to inform a proactive paradigm of individualised care and early intervention, whilst emphasising the importance of NBS to optimise functional gains, independence and participation.

Results of this study attest to the high levels of adaptive functioning that is possible for children with SMA who have been treated with DMTs, which is an important finding for families who emphasise a strengths-based approach to care and support. Collective strengths across the cohort (independent of modality of diagnosis) include social/cognitive and responsibility aspects of adaptive functioning. Identifying and fostering these strengths are highly valued by the SMA community as they provide a foundation on which to promote social inclusion, interpersonal skills and independence.^3^, ^4^, ^5^

High levels of adaptive functioning were particularly striking in children diagnosed through clinical referral, where significantly lower performance-based measures (in comparison to those diagnosed through NBS) did not necessarily translate to differences in (responsibility) components of adaptive functioning. A crucial element of adaptive functioning is independent movement, achieved through ambulation or assistive mobility devices.^21^ Our findings suggest that adaptive functioning may be facilitated by the presence of supportive environmental factors including assistive technologies (e.g., lower limb orthoses and wheelchairs) and school supports (e.g., aides to assist with self-care tasks) to overcome functional challenges.

Whilst the international evidence base denotes that NBS for SMA is a key modifier of motor function outcomes, here we determine that early identification of SMA through NBS and timely intervention with DMTs can also significantly change the trajectory of adaptive functioning from the perspective of every day practical tasks and activities of daily living.^2^ Our findings also highlighted that regardless of diagnostic modality, children with DMT-treated SMA commonly encountered difficulties with independent standing, transfers, and navigating stairs. Furthermore, a high proportion of CR children and NBS children with 2 *SMN2* copies used mobility supports for travelling extended distances, including lower limb orthoses and manual/power wheelchairs. Synthesising these caregiver-reported findings with performance-based measurements of motor function, notably the 6MWT, may suggest that physical fatigue is an enduring element of SMA that impedes adaptive functioning, aligning with findings from prior adult studies.^22^ Moreover, these findings provide a broader perspective of motor function, attesting to the effect of NBS for school-aged children in everyday life and higher motor tasks beyond independent walking. Implementing tailored environmental modifications at school and within the community is crucial to mitigate fatigue and bolster participation; for example, fatigue management strategies, use of mixed mobility for extended distances, minimising stairs, placing classrooms within proximity and utilising accessible mobility parking permits in the community.^23^ Furthermore, future treatments focussed on the amelioration of muscle fatigue measured using person-reported and performance-based outcomes are essential to minimise the effects of this symptom upon adaptive functioning.^24^

The ICF framework facilitates connections between performance-based measures and caregiver responses, highlighting the benefit of utilising both outcomes concurrently. For example, the 6MWT assesses endurance (body structures and function domain), however the fatiguability identified through this assessment affected children's adaptive functioning and community ambulation (activity and participation domains).^24^ Applying a consistent framework to assess adaptive functioning that is meaningful and relevant to the needs of affected children is critical to navigate the intricacies of therapeutic goal-setting, further complicated by treatments and the cycles of hope and distress experienced by families.^25^ A meta-analysis investigating children with cerebral palsy demonstrated that interventions and treatment goals aligned with everyday participation as per the ICF are more effective than those which only consider activities or body functions and structures.^26^ Additionally, the ICF coding system has been applied in a pilot study for children with SMA who received DMTs, establishing that implementation of the ICF is an important step for holistic management of children with SMA in the era of DMTs.^27^ Thus, the clinical paradigm of monitoring for all children with SMA should include a proactive stance on multidimensional assessment of adaptive functioning through caregiver and performance-based measures within an interdisciplinary model of care. As applications of the ICF framework to SMA care and rehabilitation models expands, ongoing participatory research is crucial to operationalise the ICF framework effectively in real-world settings. This engagement can further shape development, validation and implementation of SMA outcome measures for clinical research and real-world evaluation of treatment efficacy.

Prior to the advent of DMTs, high levels of caregiver stress were reported amongst caregivers of children with SMA, associated with the social, psychological and financial stressors of caregiving.^28^ Our study demonstrated that while a range of stress severity was reported, no caregivers reported elevated scores over 55; aligning with findings from prior studies amongst caregivers of children with early onset SMA treated with DMTs, who demonstrated resilience and positive coping strategies despite anxiety associated with caregiving.^6^ Furthermore, the similar levels of stress reported between the cohorts suggest that psychosocial supports and tailored interventions to improve emotional and mental wellbeing for caregivers of children with SMA are important regardless of modality of diagnosis. Recent evidence has emphasised that a lack of psychosocial and psychological support networks exists for caregivers whose children have received DMTs for SMA, which compounds caregiver burden; reinforcing the imperative for interventions to optimise caregiver wellbeing, and thus functional outcomes for children with SMA.^29^

The strengths of this study include the robust study response rate, inclusion of NBS and clinically referred children to provide generalisability to different healthcare settings and the fact that this cohort was prospectively evaluated through a multidimensional assessment framework, integrating performance, functional and family perspectives to provide a holistic view of the modifiers and enablers of adaptive functioning. This study denotes adaptive functioning in the first international cohorts of children diagnosed through NBS as they reach school age, with the long duration of follow up providing evidence for the strengths and challenges children encounter. By using a framework that interrogates adaptive functioning through its component parts, the study findings represent a move towards integrating the holistic needs of the child and views of the family into healthcare assessment, which is highly valued by the SMA community.^3^

Variation in baseline characteristics between the NBS and clinically referred groups was a limitation to the study. This is due to children in the NBS group being younger and a greater proportion predicted to develop early severe SMA. However, the study findings are magnified in this context considering that the NBS group demonstrated higher motor function than their chronologically older clinically referred counterparts. Furthermore, the usefulness of the PEDI-CAT varied across the population, with low fit scores in the mobility domain of the PEDI-CAT indicative of a floor effect amongst non-ambulant children in the clinically referred group. This aligns with previous literature in which Rasch analysis found floor effects occurred in early-onset SMA.^11^ Thus, while PEDI-CAT was useful in characterising broad functional abilities and was appreciated by caregivers to provide useful information, the tool could not capture small functional changes in these groups. Cognition, social behaviours, communication and language are important elements of adaptive functioning which can directly impact everyday participation, and differences in these aspects are emerging for children with SMA type I.^30^ Whilst most children scored within the expected range for the social/cognitive PEDI-CAT domain, the tested content areas focussed on self-management, interaction, communication and everyday cognition, which is a limitation of the primary outcome measure used within this study. Additional speech and language assessments will enhance findings, yet require validation to determine whether differences are attributable to motor weakness, fatigue related impairments or associated neurodevelopmental comorbidities.

The trajectory of children with SMA who have received DMTs is emerging in real-time, and this study provides an understanding of their real-world outcomes. Whilst all children in the cohort were treated, future work may focus on evaluation of DMT type as a modifier of adaptive functioning. Incorporating a multidisciplinary neurorehabilitative model of care is essential to meet the changing and individualised needs of children with SMA, especially as they transition to complete higher motor tasks and complex everyday activities when participating at home, school and within the community. This approach allows clinicians to elucidate areas of functional strength and weakness, thereby enriching interventions, mitigating distress and optimising adaptive functioning, as seen in other neurodevelopmental conditions.

## Contributors

E.E. Farah: conceptualisation, methodology, investigation, data curation, formal analysis, writing—original draft, writing—review and editing. SG. Paguinto: conceptualisation, methodology, investigation, formal analysis, writing—review and editing. M.R. Mandarakas: investigation, data curation, formal analysis, writing—review and editing. K. Herbert: conceptualisation, investigation, writing—review and editing. S. Krishan: investigation, writing—review and editing. H. Sampaio: investigation, writing—review and editing. D.K. Kariyawasam: conceptualisation, formal analysis, funding acquisition, investigation, methodology, writing—original draft, writing—review and editing. M.A. Farrar: conceptualisation, formal analysis, funding acquisition, investigation, methodology, writing—original draft, writing—review and editing. E.E. Farah and M.R. Mandarakas have directly accessed and verified the underlying data in the manuscript. E.E. Farah and M.A. Farrar were responsible for the decision to submit the manuscript.

## Data sharing statement

Data collected in the study may be made available to appropriately qualified researchers for reasonable requests. Data provided will be de-identified participant data. Applications should be submitted 1–12 months following publication of the article in print, and should have accompanying evidence of approval from an independent review committee for specific use of the data. This application should be submitted to the corresponding author who may share the de-identified data with a signed data access agreement.

## Editor note

This translation in Arabic and Chinese was submitted by the authors and we reproduce it as supplied. It has not been peer reviewed. Our editorial processes have only been applied to the original abstract in English, which should serve as reference for this manuscript.

## Declaration of interests

M.A. Farrar has received honoraria for scientific advisory boards and educational activities from Novartis Gene Therapies, Inc., Biogen, and Roche, and research grants from NHRMC; D.S. Kariyawasam has received honoraria for scientific advisory boards and educational activities from Novartis Gene Therapies, Inc., Biogen, and Roche, and research grants from NHMRC. S.G. Paguinto has received honoraria for educational event participation from Biogen. E.E. Farah received funding support from a Univeristy of New South Wales Scholarship. M.R. Mandarakas, K. Herbert, S. Krishan and H. Sampaio report no disclosures relevant to the manuscript.

## Appendix A Supplementary data

Appendix

Abstract Arabic

Abstract Chinese

## References

[1] Mercuri E., Sumner C.J., Muntoni F., Darras B.T., Finkel R.S. (2022). Spinal muscular atrophy. Nat Rev Dis Primers.

[2] Kariyawasam D.S., D'Silva A.M., Sampaio H. (2023). Newborn screening for spinal muscular atrophy in Australia: a non-randomised cohort study. Lancet Child Adolesc Health.

[3] de Lemus M., Cattinari M.G., Pascual S.I. (2024). Identification of the most relevant aspects of spinal muscular atrophy (SMA) with impact on the quality of life of SMA patients and their caregivers: the PROfuture project, a qualitative study. J Patient-Rep Outcomes.

[4] Mongiovi P., Dilek N., Garland C. (2018). Patient reported impact of symptoms in spinal muscular atrophy (PRISM-SMA). Neurology.

[5] McGraw S., Qian Y., Henne J., Jarecki J., Hobby K., Yeh W.-S. (2017). A qualitative study of perceptions of meaningful change in spinal muscular atrophy. BMC Neurol.

[6] Tosi M., Cumbo F., Catteruccia M. (2023). Neurocognitive profile of a cohort of SMA type 1 pediatric patients and emotional aspects, resilience and coping strategies of their caregivers. Eur J Paediatr Neurol.

[7] Lee Y.J., Kim A.R., Lee J.-M. (2023). Impact of nusinersen on the health-related quality of life and caregiver burden of patients with spinal muscular atrophy with symptom onset after age 6 months. Muscle Nerve.

[8] Jiang L., Yan Y., Yu Y. (2025). Impact of nusinersen treatment on the independence and mental health of school-aged patients with spinal muscular atrophy. Pediatr Neurol.

[9] World Health Organization (2001).

[10] (Suppl 2006). WHO Motor development study: windows of achievement for six gross motor development milestones. Acta Paediatr.

[11] Pasternak A., Sideridis G., Fragala-Pinkham M. (2016). Rasch analysis of the pediatric evaluation of disability Inventory–computer adaptive test (PEDI-CAT) item bank for children and young adults with spinal muscular atrophy. Muscle Nerve.

[12] Thompson S.V., Cech D.J., Cahill S.M., Krzak J.J. (2018). Linking the pediatric evaluation of disability inventory-computer adaptive test (PEDI-CAT) to the international classification of function. Pediatr Phys Ther.

[13] Fragala-Pinkham M., Pasternak A., McDermott M.P. (2021). Psychometric properties of the PEDI-CAT for children and youth with spinal muscular atrophy. J Pediatr Rehabil Med.

[14] Pera M.C., Coratti G., Forcina N. (2017). Content validity and clinical meaningfulness of the HFMSE in spinal muscular atrophy. BMC Neurol.

[15] Mazzone E.S., Mayhew A., Montes J. (2017). Revised upper limb module for spinal muscular atrophy: development of a new module. Muscle Nerve.

[16] Dunaway S., Montes J., Garber C.E. (2014). Performance of the timed "up & go" test in spinal muscular atrophy. Muscle Nerve.

[17] Dunaway Young S., Montes J., Kramer S.S. (2016). Six-minute walk test is reliable and valid in spinal muscular atrophy. Muscle Nerve.

[18] McKay M.J., Baldwin J.N., Ferreira P., Simic M., Vanicek N., Burns J. (2017). Reference values for developing responsive functional outcome measures across the lifespan. Neurology.

[19] Pasternak A., McDermott M.P., Montes J. (2025). Spinal muscular atrophy functional composite score revised (SMA-FCR) in untreated and nusinersen-treated patient cohorts. Neurology.

[20] Berry J.O., Jones W.H. (1995). The parental stress scale: initial psychometric evidence. J Soc Pers Relat.

[21] Palisano R.J., Chiarello L.A., King G.A., Novak I., Stoner T., Fiss A. (2012). Participation-based therapy for children with physical disabilities. Disabil Rehabil.

[22] Parsons J.A., Land N., Maravic M.C. (2025). Remaining burden of spinal muscular atrophy among treated patients: a survey of patients and caregivers. Ann Clin Transl Neurol.

[23] Milićević M. (2023). Functional and environmental predictors of health-related quality of life of school-age children with cerebral palsy: a cross-sectional study of caregiver perspectives. Child Care Health Dev.

[24] Crisafulli O., Berardinelli A., D'Antona G. (2024). Fatigue in spinal muscular atrophy: a fundamental open issue. Acta Myol.

[25] Paguinto S.G., Kasparian N.A., Bray P., Farrar M. (2020). "It's not just the wheelchair, it's everything else": Auatralian parents' perspectives of wheelchair prescription for children with neuromuscular disorders. Disabil Rehabil.

[26] Reedman S., Boyd R.N., Sakzewski L. (2017). The efficacy of interventions to increase physical activity participation of children with cerebral palsy: a systematic review and meta-analysis. Dev Med Child Neurol.

[27] Giannotta G., Ruggiero M., Pirani G., Oliva M.C., Ferrante C., Trabacca A. (2025). Setting multidisciplinary intervention goals for spinal muscular atrophy patients utilizing the international classification of functioning, disability, and health: a pilot study in a small sample sizes. Acta Neurol Belg.

[28] Farrar M.A., Carey K.A., Paguinto S.G., Chambers G., Kasparian N.A. (2018). Financial, opportunity and psychosocial costs of spinal muscular atrophy: an exploratory qualitative analysis of Australian carer perspectives. BMJ Open.

[29] Landfeldt E., Udo C., Cortina-Borja M., Sejersen T., Kreicbergs U. (2025). "Not only has she survived, but she lives a happy life": parents' perspectives and experiences of a novel disease-modifying therapy for spinal muscular atrophy in Sweden. J Child Neurol.

[30] Buchignani B., Coratti G., Cutrì C. (2025). Neurodevelopmental and mental disorders in children with type I and presymptomatic spinal muscular atrophy. Sci Rep.

