# Supplementary material for: Adaptive functioning in school-aged children with spinal muscular atrophy in the treatment era: a non-randomised cohort study
Source: Lancet Reg Health West Pac. 2026 Apr 30;70:101866. doi: 10.1016/j.lanwpc.2026.101866 (PMC13146543; doi:10.1016/j.lanwpc.2026.101866)
Supplement: Appendix [file mmc1.docx]

**eAppendix – Clinical Consult Questionnaire**

**Equipment**

| Equipment used at home:  🞏 Walking aids (crutches, walker)  🞏 Trunk brace  🞏 Wheelchair (manual)  🞏 Wheelchair (power)  🞏 Comments:  ___________________________________  ____________________________________ | Equipment used at school:  🞏 Walking aids (crutches, walker)  🞏 Ankle-foot orthoses  🞏 Trunk brace  🞏 Wheelchair (manual)  🞏 Wheelchair (power)  🞏 Comments:  _______________________________________  _______________________________________ |
| --- | --- |

**Self-Care**

| Toileting:  🞏 Independent, no equipment  🞏 Independent, with equipment  🞏 Needs physical assistance  🞏 Comments:  ___________________________________  ____________________________________ | Showering and/or bathing:  🞏 Independent, no equipment  🞏 Independent, with equipment  🞏 Needs physical assistance  🞏 Comments:  _______________________________________  ______________________________________ |
| --- | --- |
| Feeding:  🞏 Independent (oral feeding)  🞏 Physical assistance  🞏 Modified diet  🞏 PEG  🞏 Comments:  ____________________________________  ____________________________________ | Dressing:  🞏 Independent  🞏 Needs prompting  🞏 Needs equipment (e.g., dressing aids, modified clothing)  🞏 Needs physical assistance  🞏 Comments:  ______________________________________  _____________________________________ |
| *What are the specific self-care goals that the child and family are working towards?*  ___________________________________________________________________________ | |
| **School** **Supports**:  🞏 School Learning Support Officer  🞏 Assistive technology  🞏 Completed and implemented Individual Learning Plan  🞏 Other (please detail):________________________________  🞏 Comments:  *What hand function skills can the child perform well (e.g., writing, scissors)? What are some goals they are working towards?*  ___________________________________________________________________________ | |
