# Supplementary material for: Adaptive functioning in school-aged children with spinal muscular atrophy in the treatment era: a non-randomised cohort study
Source: Lancet Reg Health West Pac. 2026 Apr 30;70:101866. doi: 10.1016/j.lanwpc.2026.101866 (PMC13146543; doi:10.1016/j.lanwpc.2026.101866)
Supplement: Abstract Arabic [file mmc2.docx]

**الأداء التكيفي لدى الأطفال في سن المدرسة الذين يعانون من ضمور العضلات الشوكي في عصر العلاجات الحديثه: دراسة جماعية غير عشوائية**

**الخلاصه**

**الخلفية**: على الرغم من التقدم التشخيصي والعلاجي لضمور العضلات الشوكي (SMA)، فإن النتائج بعيدة المدى المتعلقة بالاستقلالية الوظيفية لدى الاطفال المصابين لم تدرس بشكل كاف.تهدف هذه الدراسة إلى توصيف مستوى الأداء التكيفي للأطفال المصابين بالضمور العضلي الشوكيّ في ظل نموذج العلاج المعاصر.

**الأساليب**:

أجريت هذه الدراسه الأترابية المستقبلية غير العشوائية في استراليا خلال الفتره من 1 يناير إلى 14 نوفمبر 2025 وشملت الدراسه الأطفال المصابين ب الضمور العضلي الشوكي الذين تتراوح أعمارهم بين 4 و 12 عاما، تم تشخيصهم وعلاجهم من خلال فحص الأطفال حديثي الولادة (NBS) أو الإحالة السريرية (CR). تم تقييم الأداء التكيفي بشكل رئيسي باستخدام إختبار التقيم الحاسوبي ( قائمة تقيم الاعاقه لدى الأطفال).

**النتائج**:

شارك تسعة وثلاثون طفلًا في الدراسة (الفحص حديثي الولادة NBS: عدد = 18، والإحالة السريرية CR: عدد = 21)، بمتوسط مدة علاج وسيطي بلغ 67.3 شهرًا (المدى الربيعي IQR: ‏54.0–85.0 شهرًا). أظهر الأطفال مستويات مرتفعة من المشاركة، حيث كانت الدرجات ضمن النطاق المتوقع مقارنةً بالأقران المماثلين في العمر في مجالات التفاعل الاجتماعي/المعرفي (38 من 39؛ ‎97%‎)، وتحمّل المسؤولية (35 من 39؛ ‎90%‎)، والأنشطة اليومية (29 من 39؛ ‎74%‎)، والحركة والتنقّل (16 من 39؛ ‎41%‎).

كما أظهرت نسبة أكبر من الأطفال الذين تم تشخيص حالتهم عبر برنامج الفحص لحديثي الولادة (NBS) تحقيق درجات متوقعة في جميع المجالات مقارنةً بالأطفال الذين تم تشخيصهم عبر الإحالة السريرية (CR) (‏14 من 18؛ ‎78%‎ مقابل ‏2 من 21؛ ‎10%‎؛ ‏p < 0.001).

وحقق جميع الأطفال الذين شُخِّصوا عبر برنامج الفحص لحديثي الولادة والذين لديهم ثلاث نسخ من جين SMN2 درجات ضمن النطاق المتوقع في جميع المجالات (3 نسخ SMN2: ‏7/7؛ ‎100%‎)، مقارنةً بالأطفال الذين لديهم نسختان من الجين SMN2 (‏7 من 11؛ ‎64%‎).

أما الأطفال الذين شُخِّصوا عبر الإحالة السريرية (CR)، فقد أظهر أولئك الذين كانت لديهم حالة وظيفية أعلى عند التشخيص تواترًا أكبر لدرجات الأنشطة اليومية ضمن النطاق المتوقع (القادرون على المشي: ‏7/7؛ ‎100%‎، الجالسون: ‏2/5؛ ‎40%‎، غير القادرين على الجلوس: ‏3/9؛ ‎33%‎؛ ‏p = 0.02).

**التفسير**:

على الرغم من وجود تباين في مقاييس الأداء التكيفي بين الأطفال، فإن الأطفال المصابين بـضمور العضلات الشوكي (SMA) يُظهرون بشكل جماعي نقاط قوة في الجوانب الاجتماعية-المعرفية وجوانب تحمّل المسؤولية ضمن الوظائف التكييفية. كما يُعد التشخيص المبكر والعلاج من خلال برنامج فحص حديثي الولادة (NBS)، إضافةً إلى عدد نسخ جين SMN2، من العوامل المعدِّلة المهمة للوظيفة التكيفية على المدى الطويل. وتشير هذه النتائج إلى أهمية وضع أهداف علاجيه فردية، والمتابعة المستمرة، وتقديم رعاية متعددة التخصصات.
