# Supplementary material for: Adaptive functioning in school-aged children with spinal muscular atrophy in the treatment era: a non-randomised cohort study
Source: Lancet Reg Health West Pac. 2026 Apr 30;70:101866. doi: 10.1016/j.lanwpc.2026.101866 (PMC13146543; doi:10.1016/j.lanwpc.2026.101866)
Supplement: Abstract Chinese [file mmc3.docx]

**Traditional Chinese**

治療時代 - 學齡期脊髓性肌肉萎縮症患兒的適應性功能水平：一項非隨機隊列研究

【摘要】

**目的**：探討在目前已有的治療模式下，脊髓性肌肉萎縮症 （Spinal Muscular Atrophy，SMA） 患兒的適應性功能水平。

**方法**：本研究為前瞻性非隨機隊列研究，於2025年1月1日至2025年11月14日在澳大利亞開展，並招攬了4至12歲的SMA患兒，根據確診和接受治療的方式，將他們分為新生兒篩查組 （Newborn Screening，NBS） 或臨床轉介組（Clinical Referral，CR）。 是次採用了兒童殘障評估量表計算機自適應測試 （Pediatric Evaluation of Disability Inventory Computer Adaptive Test），對患兒的適應性功能進行評估。

**結果**：共納入39例SMA患兒（NBS組 18例，CR 組21例），他們的治療中位時間為67.3個月（四分位距54.0-85.0個月）。患兒於社會/認知（38/39，97%）、責任感（35/39，90%）、日常生活活動（29/39，74%）和行動能力（16/39，41%）等領域的得分在同齡兒童的預期分數範圍內。與CR 組相比，NBS組患兒在所有功能領域達到預期分數的比例更高（NBS 14/18 (78%)，CR 2/21 (10%)，p＜0.001）。所有透過NBS確診，並攜帶 3 個運動神經元存活基因 2 (SMN2) 拷貝的患兒，在所有領域均達到了預期分數（3 個 SMN2 7/7 (100%)，2 個 SMN2 7/11 (64%)）。比起NBS組，CR組確診時功能水平較高的患兒，在日常生活活動領域中達到預期分數的比例更高（可行走者7/7 (100%)，可坐者2/5 (40%)，不能坐者3/9 (33%)， p = 0.02）。

**結論**：儘管SMA患兒在表現型測驗有一定的異質性，但在社會認知及責任感相關領域整體表現良好。NBS所帶來的早期診斷與治療以及SMN2基因拷貝數是影響兒童長期適應性功能的重要因素。是次研究印證了這兩個因素對於為兒童設立個人化目標、實行醫學監測和設立多學科照護有一定的影響性。

**Simplified Chinese**

治疗时代 - 学龄期脊髓性肌肉萎缩症患儿的适应性功能水平：一项非随机队列研究

【摘要】

**目的**：探讨在目前已有的治疗模式下，脊髓性肌肉萎缩症 （Spinal Muscular Atrophy，SMA） 患儿的适应性功能水平。

**方法**：本研究为前瞻性非随机队列研究，于2025年1月1日至2025年11月14日在澳大利亚开展，并招揽了4至12岁的SMA患儿，根据确诊和接受治疗的方式，将他们分为新生儿筛查组 （Newborn Screening，NBS） 或临床转介组（Clinical Referral，CR）。 是次采用了儿童残障评估量表计算机自适应测试 （Pediatric Evaluation of Disability Inventory Computer Adaptive Test），对患儿的适应性功能进行评估。

**结果**：共纳入39例SMA患儿（NBS组 18例，CR 组21例），他们的治疗中位时间为67.3个月（四分位距54.0-85.0个月）。患儿于社会/认知（38/39，97%）、责任感（35/39，90%）、日常生活活动（29/39，74%）和行动能力（16/39，41%）等领域的得分在同龄儿童的预期分数范围内。与CR 组相比，NBS组患儿在所有功能领域达到预期分数的比例更高（NBS 14/18 (78%)，CR 2/21 (10%)，p＜0.001）。所有透过NBS确诊，并携带 3 个运动神经元存活基因 2 (SMN2) 拷贝的患儿，在所有领域均达到了预期分数（3 个 SMN2 7/7 (100%)，2 个 SMN2 7/11 (64%)）。比起NBS组，CR组确诊时功能水平较高的患儿，在日常生活活动领域中达到预期分数的比例更高（可行走者7/7 (100%)，可坐者2/5 (40%)，不能坐者3/9 (33%)，p = 0.02）。

**结论**：尽管SMA患儿在表现型测验有一定的异质性，但在社会认知及责任感相关领域整体表现良好。 NBS所带来的早期诊断与治疗以及SMN2基因拷贝数是影响儿童长期适应性功能的重要因素。是次研究印证了这两个因素对于为儿童设立个人化目标、实行医学监测和设立多学科照护有一定的影响性。
