# Supplementary material for: Adaptive functioning in school-aged children with spinal muscular atrophy in the treatment era: a non-randomised cohort study
Source: Lancet Reg Health West Pac. 2026 Apr 30;70:101866. doi: 10.1016/j.lanwpc.2026.101866 (PMC13146543; doi:10.1016/j.lanwpc.2026.101866)
Supplement: Supplementary Figures and Tables [file mmc4.docx]

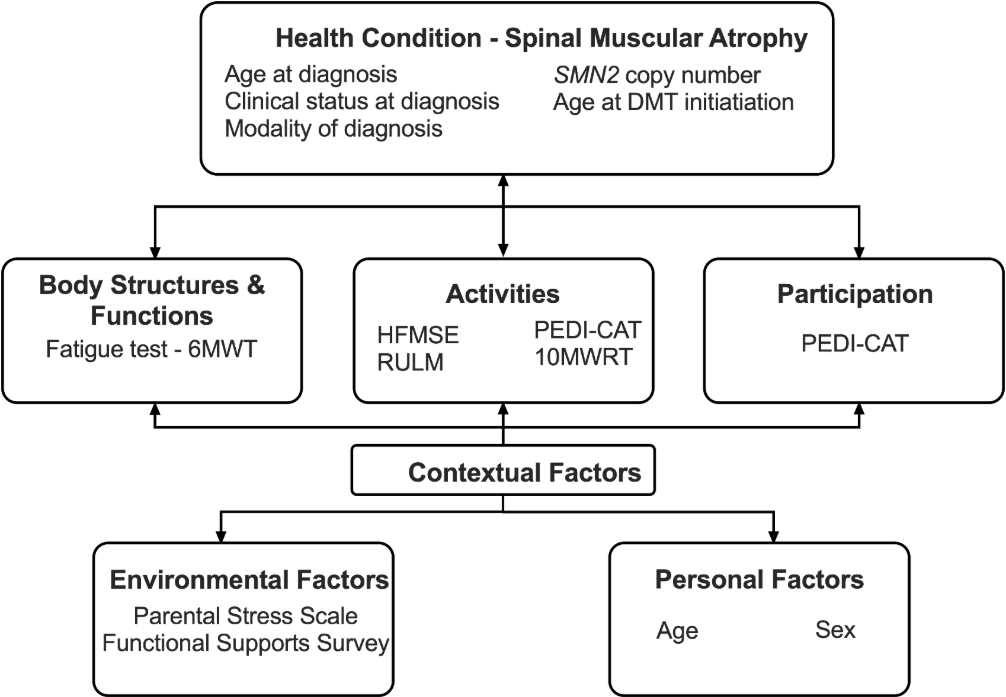


**Supplementary Figure 1: Study measures as aligned within the International Classification of Functioning, Disability and Health Framework.**

Adapted from the World Health Organization.^9^ Abbreviations: HFMSE, Hammersmith Functional Motor Scale – Expanded; PEDI-CAT, Pediatric Evaluation of Disability Inventory Computer Adaptive Test; RULM, Revised Upper Limb Module; *SMN2,* Survival Motor Neuron 2 gene; 6MWT, Six-Minute Walk Test; 10MWRT, Ten-Metre Walk/Run Test.


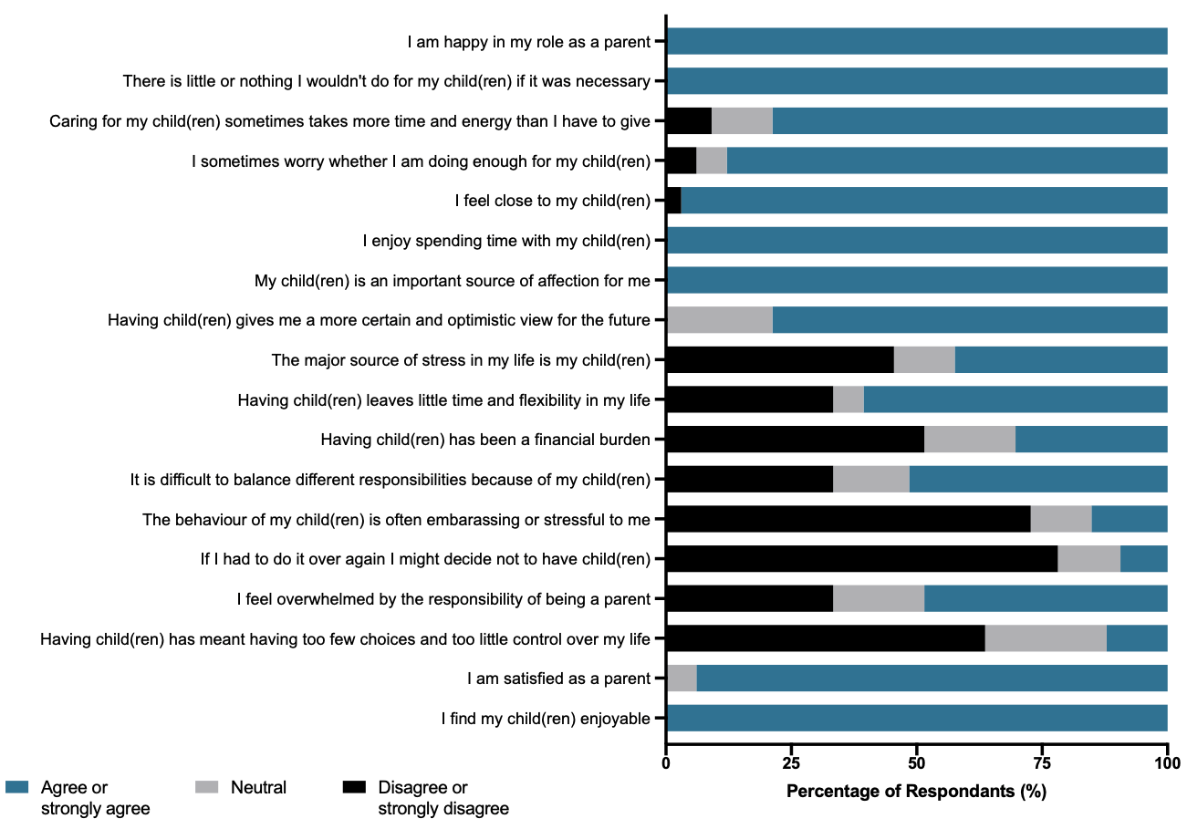


**Supplementary Figure 2: Parental Stress Survey (PSS) responses as reported by caregivers of children with SMA.**Caregiver (n=33) responses to each prompt were reported as agree or strongly agree (blue), neutral (grey), and disagree or strongly disagree (black).

**Supplementary Table 1: Motor function scores for** **children with SMA by modality of diagnosis**

|  | **Newborn Screening Group (n=18)** | **Clinically Referred Group (n=21)** | **p value** |
| --- | --- | --- | --- |
| **HFMSE** | | | |
| Median (IQR) | 63·0 (51·0 – 65·5) | 38·0 (14·0 – 57·5) | 0·001 |
| Range | 38·0 – 66·0 | 2·0 – 66·0 |  |
| **RULM Dominant Hand** | | | |
| Median (IQR) | 30 (27 – 36) | 28 (21 – 37) | 0·623 |
| Range | 23 – 37 | 11 – 37 |  |
| **6MWT (metres)** | | | |
| Median (IQR) | 350·0 (236 – 417) | 0 (0 – 323) | 0·003 |
| Range | 0 – 32 | 0 – 84 |  |
| **Predicted 6MWT** | | | |
| Median (IQR) | 75·8 (48·4 – 87·6) | 0 (0 – 56·2) | <0·001 |
| Range | 0 – 97·8 | 0 – 77·5 |  |
| **SMA-FCR** | | | |
| Median (IQR) | 79·8 (68·5 – 93·3) | 45·1 (29·5 – 85·4) | 0·002 |
| Range | 51·8 – 96·3 | 17·3 – 91·1 |  |

Children (n=39)* were identified through newborn screening or clinical referral. Children unable to stand or initiate the 6MWT scored “0”. *All motor function measures were collated for 34 children - 2 children could not comply with the performance-based assessments, RULM missing for 3 children due to resource limitations. Abbreviations: HFMSE, Hammersmith Functional Motor Scale – Expanded; IQR, Interquartile Range; RULM, Revised Upper Limb Module; SMA; spinal muscular atrophy; SMA-FCR, Spinal Muscular Atrophy Functional Composite Score Revised; 6MWT, Six-Minute Walk Test.

**Supplementary Table 2: Motor function scores and timed tests for** **children with SMA diagnosed through NBS by copy number**

|  | **2 SMN2** | **3 SMN2** | **p value** |
| --- | --- | --- | --- |
| **HFMSE** | | | |
| Median (IQR) | 51.5 (47.0 – 60.0) | 65·0 (64·0 – 66·0) | 0·004 |
| Range | 38·0 – 66·0 | 63·0 – 66·0 |  |
| **RULM Dominant Hand** | | | |
| Median (IQR) | 28·0 (26·0 – 30·5) | 35·0 (32·25 – 36·25) | 0·018 |
| Range | 23·0 – 37·0 | 30·0 – 37·0 |  |
| **6MWT (metres)** | | | |
| Median (IQR) | 280·5 (125·0 – 365·0) | 412·0 (397·0 – 475·0) | 0·011 |
| Range | 0 – 461·0 | 301·0 – 532·0 |  |
| **Predicted 6MWT (%)** | | | |
| Median (IQR) | 57·4 (26·2 – 77·2) | 86·1 (76·6 – 91·6) | 0·017 |
| Range | 0 – 97·8 | 62·7 – 92·2 |  |
| **SMA-FCR** | | | |
| Median (IQR) | 73·4 (62·0 – 81·4) | 93·1 (86·3 – 95·6) | 0·010 |
| Range | 52·0 – 94·0 | 80·0 – 96·0 |  |
| **10MWRT (seconds)*** | | | |
| Median (IQR) | 7·1 (5·8 – 12·9) | 4·5 (4·3 – 5·5) | 0·007 |
| Range | 3.8 – 17.5 | 3·6 – 5·6 |  |

Children (n=18)* were identified through newborn screening. *All motor function measures were collated for 15 children - 2 children with 2 *SMN2*copies could not comply with the performance-based assessments, RULM missing for 2 children due to resource limitations. Abbreviations: HFMSE, Hammersmith Functional Motor Scale – Expanded; IQR, Interquartile Range; RULM, Revised Upper Limb Module; SMA; spinal muscular atrophy; SMA-FCR, Spinal Muscular Atrophy Functional Composite Score Revised; 6MWT, Six-Minute Walk Test; 10MWRT, Ten-Metre Walk/Run test.
